# Co-Designing and Evaluating a Self-Care Curriculum of an App for Chinese Immigrant Caregivers

**DOI:** 10.1093/geroni/igaa057.1639

**Published:** 2020-12-16

**Authors:** Ying Wang, Mandong Liu, Iris Chi

**Affiliations:** 1 Lanzhou University, Lanzhou, Gansu, China; 2 University of Southern California, Los Angeles, California, United States

## Abstract

Chinese immigrant caregivers face unique self-care difficulties in the United States due to language barriers, cultural isolation, and occupational stress. This study aimed to conduct a formative evaluation on a caregiver self-care curriculum of an app designed for Chinese immigrants in the United States. Using a co-design approach in 2019, 22 Chinese immigrant caregivers in Los Angeles county were recruited through purposive sampling method. The directed content analysis was adopted to analyze the qualitative data using NVivo 12.1.0 software. We organized the findings under two main contents: self-care and caregiving. Three categories were identified under the self-care content: physical health, emotional and mental health, and support resources. Sixteen subcategories under physical health (e.g., dietary supplements), five subcategories under emotional and mental health (e.g., depression) and eight subcategories under support resources (e.g., support and networking group, senior center) are suggested. Two categories were identified under the caregiving content: caregiving knowledge and skills, and community resources. Fourteen subcategories under caregiving knowledge and skills (e.g., care assessment) and six subcategories under community resources (e.g., medical emergency call) were mentioned. With this useful information, we could further refine the self-care curriculum to be more linguistically, culturally and occupationally sensitive for Chinese immigrant caregivers. Empowerment approach for enhancing the ability to caregiving and self-care should be emphasized in content design for immigrant caregivers. The co-design approach is crucial for planning of the program and intervention curriculum to improve understanding of the users’ needs and better cater them.

